# Satisfactory Short-Term Outcomes of Reverse Shoulder Arthroplasty for Complex Three- and Four-Part Fractures of the Humeral Head in Octogenarians

**DOI:** 10.7759/cureus.53604

**Published:** 2024-02-05

**Authors:** Ioannis Gigis, Theofylaktos Kyriakidis, Triantafyllos Katsimentzas, Alexandros Nenopoulos, Roderich Heikenfeld, Konstantinos Ditsios, Periklis Papadopoulos

**Affiliations:** 1 2nd Orthopaedic Department, General Hospital "G. Gennimatas" Aristotle University of Thessaloniki, Thessaloniki, GRC; 2 Center for Orthopaedics and Traumatology, St. Elisabeth Group - Catholic Hospitals Rhein-Ruhr, Herne, DEU

**Keywords:** clinical outcomes, humeral head fracture, octogenarians, elderly, reverse shoulder arthroplasty

## Abstract

Background: Proximal humeral fractures with severe comminution and poor bone quality are among the most common injuries in the elderly population. Reverse shoulder arthroplasty (RSA) has been widely used to manage complex three- and four-part humeral head fractures. The purpose of the present study was to report the result of this technique in the demanding population of octogenarians.

Materials and methods: Twenty-six patients above the age of 80 years were included in the study and followed for a minimum of one-year follow-up. To assess the functional outcomes the postoperative range of motion (ROM), the Constant score, the visual analog scale for pain, and the disability of the arm and shoulder score (DASH) were measured at 6 and 12 months. Radiological assessment and potential complications were also recorded.

Results: The mean age of the study population was 81.9 years (81-86) at the time of surgery. There was a statistically significant improvement in all outcomes over the follow-up intervals. Shoulder ROM was 125.7^o^ for flexion, 98.2^o^ for abduction, 42.2^o^ for internal rotation, and 43.2^o^ for external rotation at 12 months. The mean Constant, DASH, and VAS scores at the last follow-up were 61.3, 31.9, and 0.5, respectively. Reported complications include one superficial surgical site infection.

Conclusion: RSA is a safe and reliable surgical option with satisfactory outcomes to manage complex three- and four-part fractures of the humeral head as it can provide prompt pain relief and function in octogenarians.

## Introduction

Proximal humeral fractures are among the most common injuries in the population above the age of 65 years [1,2]. Often, it occurs as a result of low-energy trauma, such as falls onto the outstretched limb or the shoulder itself [3,4].

The increase in life expectancy and activity level affects the number of such fractures that have constantly risen in the last decades. Such fractures require a highly challenging treatment especially when a poor-quality bone is present [5].

Indeed, the association with osteoporosis, potential age-linked rotator cuff degeneration, and multiple comorbidities make surgical treatment complex in these patients [6]. Several treatment modalities have been described to cope with this significant issue ranging from conservative treatment to surgery, including osteosynthesis with plate or nail, hemiarthroplasty, or reverse shoulder arthroplasty (RSA) [7-10].

The latter was initially introduced to treat glenohumeral arthritis with rotator cuff arthropathy; however, it is widely used for other indications such as rotator cuff deficiency, chronic dislocations, or proximal humeral fractures [11-13].

RSA is an alternative treatment for complex three- and four-part proximal humeral fractures in the elderly population, providing adequate deltoid elongation and decreasing the forces required to abduct the arm by medializing the center of rotation, overriding the presence of a dysfunctional rotator cuff [14].

It is well documented that RSA is associated with encouraging early results and outcomes in the elderly population [15-17]. However, limited data exist in the recent literature concerning the demanding population of patients beyond 80 years.

Thus, the purpose of the present study was to investigate and report the short-term clinical, radiological, and functional outcomes of RSA for complex three- and four-part fractures in octogenarians.

## Materials and methods

This is a retrospective study with prospectively collected data. Between 2014 and 2017, 26 patients above the age of 80 years were treated with RSA for complex three- and four-part humeral head fractures based on CT scan images, and followed with a minimum of 12 months follow-up (12-48 months). Patients’ demographic characteristics are provided in Table 1. The inclusion and exclusion criteria are shown in Table 2.

**Table 1:** Patient’s demographic characteristics BMI: Body mass index; RSA: Reverse shoulder arthroplasty; ASA: American Society of Anesthesiologists

Characteristic	Patient data
Sex	Female, n=13
	Male, n=7
Age	81.9 years (range 81-86)
BMI	27.9 kg/m^2^ (range 26-34)
Side	Right, n=17
	Left, n=3
Dominant hand	17:3
Indication for RSA	Neer three-part fracture n=3
	Neer four-part fracture n=17
ASA	II n=6
	III n=13
	IV n=1

**Table 2:** Inclusion and exclusion criteria of eligibility

Inclusion criteria
1. Male and female gender with three- and four-part humeral head fracture
2. >80 years old
3. At least 12 months follow-up
Exclusion criteria
1. Previous surgery in the same shoulder
2. Neurological deficit
3. Dementia
4. >2 weeks after trauma

All procedures were performed by two senior surgeons (IG, RH) using the SMR® (Lima Corporate, Udine, Italy) reverse total shoulder arthroplasty under general anesthesia. Patients were placed in a beach chair position, with the injured upper limb free to perform all movements. The deltopectoral approach was selected for all surgeries, and the skin incision was made from the coracoid process and extended distally and laterally to the anterior deltoid groove. The greater and lesser tuberosity were identified and mobilized. After that, non-absorbable Ethibond No. 5 sutures (Ethicon, Johnson & Johnson, New Brunswick, United States) were placed on the rotator cuff at the insertion level to the greater and lesser tuberosity. The glenoid and the humeral components were placed, the joint was reduced and checked for stability, and the tuberosities were repaired and reattached to the humerus via trans-osseous sutures and the prosthesis. The deltopectoral groove, the subcutaneous tissues, and the skin were closed.

Range of motion (ROM) was evaluated in flexion, abduction, and internal and external rotation degrees. Functional assessment tools including, the Constant Shoulder score and the disability of the arm and shoulder score (DASH), and the visual analog scale (VAS) for pain, were recorded and analyzed at 6 and 12 months. Radiological evaluation was performed with standard anteroposterior and lateral radiographs of the shoulder. Treatment-related minor and major complications were recorded. Major complications defined the cases needing revision surgery.

All patients agreed to follow the same rehabilitation protocol. The arm was placed in a sling for four weeks. Self-directed rehabilitation with pendulum exercises was allowed on day two as tolerated by the patient. No active flexion was authorized. After four weeks, the sling was removed, and a rehabilitation program with a physiotherapist began. Moderate lifting was allowed from six weeks postoperatively.

Statistical analysis was conducted using IBM SPSS Statistics for Windows, Version 24 (Released 2016; IBM Corp., Armonk, New York, United States). For all the values, the mean and standard deviation are provided. The Shapiro-Wilk test was used to check if data were normally distributed. The paired t-test for normal or the Wilcoxon signed-rank test for non-normally distributed data was performed to analyze outcomes between postoperative findings. P-values less than 0.05 were considered statistically significant.

All procedures were in accordance with the ethical standards of the Institutional Bioethics Committee and with the 1964 Helsinki Declaration and its later amendments or comparable ethical standards. Written informed consent was obtained from all subjects before the study.

## Results

Twenty-six patients who met the eligibility criteria were included in this study and followed for at least 12 months (range 12-48 months). Five patients were deceased for unrelated surgery reasons, and one minor complication was observed consisting of a superficial surgical site infection. These patients were excluded from further analysis. Statistically, significant improvement was found in clinical and functional assessment tools analyzed between the 6 and 12 months after surgery (p<0.05), as shown in Table 3. Radiographs during follow-up revealed no signs of prosthesis loosening, periprosthetic fracture, or dislocation. No major complications were recorded during the follow-up.

**Table 3:** Summary of outcomes All outcome values are described as mean and standard deviation (SD). ROM: Range of motion; DASH: Disability of the arm and shoulder score; VAS: Visual analog scale

Outcomes	6 months	12 months	p-value
ROM			
Flexion	105.2^o^ (SD: 26.68)	125.7^o^ (SD: 19.21)	p<0.05
Abduction	85.2^o^ (SD: 17.58)	98.2^o^ (SD: 17.64)	p<0.05
Internal rotation	37.5^o^ (SD: 4.44)	42.2^o^ (SD: 3.80)	p<0.05
External rotation	38.0^o^ (SD: 3.77)	43.2^o^ (SD: 3.73)	p<0.05
Constant score	46.9 (SD:13.96)	61.3 (SD: 12.76)	p<0.05
DASH	29.4 (SD: 12.67)	31.9 (SD: 12.67)	p<0.05
VAS	2.6 (SD: 2.16)	0.5 (SD: 1.00)	p<0.05

## Discussion

The present study analyses the clinical, functional, and radiological outcomes of 20 patients receiving a primary RSA after a complex humeral head fracture with a minimum follow-up of 12 months. The most important finding of the present study was that RSA is a safe and reliable surgical option with satisfactory short-term outcomes to manage complex three- and four-part fractures of the humeral head in octogenarians.

The overall outcome was satisfactory, with a significant improvement in shoulder ROM, Constant and DASH score, and VAS measurements over time. At the final follow-up, we observed a mean value of 125.7^o^ for flexion, 98.2^o^ for abduction, 42.2^o^ for internal, and 43.2^o^ for external rotation, respectively. The Constant mean score was 61.3, with a mean DASH score of 31.9 and a VAS for pain of 0.5.

These findings are in accordance with other published studies. Nevertheless, it should be noted that there is a lack of recent literature focusing on the demanding population of patients beyond the age of 80 years.

For instance, in their case series, Atıcı et al. [15] reported satisfactory results in 28 patients with a mean age of 72.5 years, treated with RSA for Neer three- and four-part or split head fracture. Postoperative ROM was similar to the present study, with a mean flexion of 130^o^, abduction of 100^o^, and external and internal rotation of 39^o^ and 40^o^, respectively. Hence, the population's higher age in the present study does not affect these clinical outcomes.

Another study conducted by Ross et al. [18] reported the results of a study population with a mean age of 79 years, which is very close to the present study. Indeed, they treated 29 humeral fractures with RSA. Again, they reported a shoulder ROM similar to the previous studies, with a mean flexion of 130^o^, abduction of 113^o^, and external rotation of 39^o^. These minimal differences in ROM are negligible for activities of daily life.

According to Kriechling et al. [19], who studied the effects of RSA on patients older than 80 years, a mean Constant-Murley score of 62 ± 15 was observed postoperatively. However, this study included mainly other indications for RSA, such as cuff tear arthropathy and isolated rotator cuff tear. The present study's Constant mean score was 61.3, including only octogenarian patients with complex humeral head fractures.

In general, studies investigating reverse shoulder prostheses for treating comminuted fractures of the proximal humerus are relatively rare, and most of these were performed in younger patients.

Cazeneuve and Cristofari [20] analyzed 36 patients treated with surgery due to proximal humerus fracture, with a mean follow-up postoperatively of 6.6 years. They had a Constant score of 53 points at last year's follow-up (72 months), reaching 67% of the mean score of the uninjured side. Villodre-Jimenez et al. [6] studied three- and four-part humeral head fractures treated with reverse arthroplasty. The sample consisted of 30 patients with a mean age of 74.9 years. The average follow-up time was 34.5 months, and the Constant score after about three years was, on average, 49.1 points.

Bufquin et al. [21] reported in a sampling of 41 patients in the last follow-up after surgery a Constant score of 44 points in contrast to 69 points in the contralateral shoulder and a DASH score of 44 points in both shoulders. The mean final follow-up was performed 22 months after the surgery. Klein et al. [22] also included 20 patients with a mean age of 74 and a follow-up of 33 months. The Constant mean score was 67.85 points, and the DASH score was 46.85. The previous studies reported their results for cohorts with a mean age of less than 80 years compared to our research. Moreover, the present study reports a similar or higher Constant score, while the DASH score was slightly lower.

Ross et al. [18] also described satisfactory clinical results for a sample of 21 patients with a mean Constant score and a mean QuickDASH score of 70.9 and 13.2 points, respectively. Another study investigating early functional outcomes was conducted by Wolfensperger et al. [23]. This prospective case series study included 33 patients aged ≥70 years. The mean Constant-Murley score was 64 points after six months of surgery and 71 points at one year postoperatively, reaching 87% compared with the contralateral shoulder. The mean DASH score reached 29 at six months and 30 at one year after follow-up.

In a recent study, Tian et al. [24] tried to assess the efficacy of RSA in proximal humerus fractures. Forty-three patients were included in this study with a mean age of 71 years. The clinical outcomes were measured in an average of 10.9 months with a mean Constant-Murley score of 88.7 points and a mean VAS score of 0.8 points. However, when evaluating the excellent results of this study, we should consider the relatively younger age group of patients compared to our sample.

There are also specific limitations that have to be considered in the current study. First, it was a case series with a small number of subjects involved. Second, there was a limited follow-up period of 12 months. However, this is acceptable in the case of patients above the age of 80 years. Third, five patients died for unrelated surgery reasons, and thus, they were excluded from further analysis.

Indeed, there is a lack of recent literature concerning the benefits of RSA for complex humeral head fractures, focusing on patients' demanding population beyond 80 years. On the other hand, the present study has various potential strengths, including the well-established study population of patients. Moreover, all surgeries were performed using the same prosthesis SMR® by two senior surgeons with vast experience in shoulder arthroplasty.

## Conclusions

RSA is a safe and reliable surgical option that can provide pain relief and satisfactory clinical and functional outcomes in octogenarians with complex three- and four-part fractures of the humeral head.

## References

[1] García-Fernández C, Lopiz Y, Rizo B, Serrano-Mateo L, Alcobía-Díaz B, Rodríguez-González A, Marco F (2018). Reverse total shoulder arhroplasty for the treatment of failed fixation in proximal humeral fractures. Injury.

[2] Schwarz AM, Hohenberger GM, Sauerschnig M (2021). Effectiveness of reverse total shoulder arthroplasty for primary and secondary fracture care: mid-term outcomes in a single-centre experience. BMC Musculoskelet Disord.

[3] Court-Brown CM, Duckworth AD, Clement ND, McQueen MM (2018). Fractures in older adults. A view of the future?. Injury.

[4] Palvanen M, Kannus P, Niemi S, Parkkari J (2006). Update in the epidemiology of proximal humeral fractures. Clin Orthop Relat Res.

[5] Kelly BJ, Myeroff CM (2020). Reverse shoulder arthroplasty for proximal humerus fracture. Curr Rev Musculoskelet Med.

[6] Villodre-Jimenez J, Estrems-Diaz V, Diranzo-Garcia J, Bru-Pomer A (2017). Reverse shoulder arthroplasty in 3 and 4 part proximal humeral fractures in patients aged more than 65 years: results and complications. Rev Esp Cir Ortop Traumatol.

[7] Giardella A, Ascione F, Mocchi M, Berlusconi M, Romano AM, Oliva F, Maradei L (2017). Reverse total shoulder versus angular stable plate treatment for proximal humeral fractures in over 65 years old patients. Muscles Ligaments Tendons J.

[8] Xie L, Ding F, Zhao Z, Chen Y, Xing D (2015). Operative versus non-operative treatment in complex proximal humeral fractures: a meta-analysis of randomized controlled trials. Springerplus.

[9] Maier D, Jaeger M, Izadpanah K, Strohm PC, Suedkamp NP (2014). Proximal humeral fracture treatment in adults. J Bone Joint Surg Am.

[10] Tuphe P, Caubriere M, Hubert L (2023). Early rehabilitation after reverse total shoulder prosthesis on fracture of proximal humerus in elderly patients provides better functional outcome. Eur J Orthop Surg Traumatol.

[11] Grammont PM, Baulot E (1993). Delta shoulder prosthesis for rotator cuff rupture. Orthopedics.

[12] Baulot E, Sirveaux F, Boileau P (2011). Grammont's idea: the story of Paul Grammont's functional surgery concept and the development of the reverse principle. Clin Orthop Relat Res.

[13] Mattiassich G, Marcovici LL, Krifter RM, Ortmaier R, Wegerer P, Kroepfl A (2013). Delta III reverse shoulder arthroplasty in the treatment of complex 3- and 4-part fractures of the proximal humerus: 6 to 42 months of follow up. BMC Musculoskelet Disord.

[14] Henninger HB, Barg A, Anderson AE, Bachus KN, Tashjian RZ, Burks RT (2012). Effect of deltoid tension and humeral version in reverse total shoulder arthroplasty: a biomechanical study. J Shoulder Elbow Surg.

[15] Atıcı T, Ermutlu C, Yerebakan S, Özyalçın A, Durak K (2021). Primary treatment of complex proximal humerus fractures using Humelock cementless reversible shoulder arthroplasty in the elderly. Ulus Travma Acil Cerrahi Derg.

[16] Grubhofer F, Wieser K, Meyer DC, Catanzaro S, Beeler S, Riede U, Gerber C (2016). Reverse total shoulder arthroplasty for acute head-splitting, 3- and 4-part fractures of the proximal humerus in the elderly. J Shoulder Elbow Surg.

[17] Rivera AR, Cardona V (2023). Reverse total shoulder arthroplasty for complex proximal humerus fracture in the elderly: clinical and radiological results. JSES Rev Rep Tech.

[18] Ross M, Hope B, Stokes A, Peters SE, McLeod I, Duke PF (2015). Reverse shoulder arthroplasty for the treatment of three-part and four-part proximal humeral fractures in the elderly. J Shoulder Elbow Surg.

[19] Kriechling P, Loucas R, Loucas M, Künzler T, Gerber C, Wieser K (2021). Primary reverse total shoulder arthroplasty in patients older than 80 years: clinical and radiologic outcome measures. J Shoulder Elbow Surg.

[20] Cazeneuve JF, Cristofari DJ (2010). The reverse shoulder prosthesis in the treatment of fractures of the proximal humerus in the elderly. J Bone Joint Surg Br.

[21] Bufquin T, Hersan A, Hubert L, Massin P (2007). Reverse shoulder arthroplasty for the treatment of three- and four-part fractures of the proximal humerus in the elderly: a prospective review of 43 cases with a short-term follow-up. J Bone Joint Surg Br.

[22] Klein M, Juschka M, Hinkenjann B, Scherger B, Ostermann PA (2008). Treatment of comminuted fractures of the proximal humerus in elderly patients with the Delta III reverse shoulder prosthesis. J Orthop Trauma.

[23] Wolfensperger F, Grüninger P, Dietrich M, Völlink M, Benninger E, Schläppi M, Meier C (2017). Reverse shoulder arthroplasty for complex fractures of the proximal humerus in elderly patients: impact on the level of independency, early function, and pain medication. J Shoulder Elbow Surg.

[24] Tian X, Xiang M, Wang G (2020). Treatment of complex proximal humeral fractures in the elderly with reverse shoulder arthroplasty. Orthop Surg.

